# Tumor-Specific CD4^+^ T Cells Restrain Established Metastatic Melanoma by Developing Into Cytotoxic CD4^–^ T Cells

**DOI:** 10.3389/fimmu.2022.875718

**Published:** 2022-06-16

**Authors:** Qiao Liu, Lisha Wang, Huayu Lin, Zhiming Wang, Jialin Wu, Junyi Guo, Shuqiong Wen, Ling Ran, Zhengliang Yue, Xingxing Su, Qing Wu, Jianfang Tang, Zhirong Li, Li Hu, Lifan Xu, Lilin Ye, Qizhao Huang

**Affiliations:** ^1^ Institute of Immunology, Third Military Medical University, Chongqing, China; ^2^ Department of Respiratory Disease, General Hospital of Xinjiang Military Command, Urumqi, China; ^3^ Guanghua School of Stomatology, Guangdong Provincial Key Laboratory of Stomatology, Stomatological Hospital, Sun Yat-sen University, Guangzhou, China; ^4^ Guangdong Provincial Key Laboratory of Immune Regulation and Immunotherapy, School of Laboratory Medicine and Biotechnology, Southern Medical University, Guangzhou, China

**Keywords:** tumor specific, CD4^+^T cells, CD4^-^T cells, metastasis, melanoma

## Abstract

Cytotoxic CD8^+^ T cells are the main focus of efforts to understand anti-tumor immunity and immunotherapy. The adoptive transfer of tumor-reactive cytotoxic CD8^+^ T lymphocytes expanded and differentiated *in vitro* has long been considered the primary strategy in adaptive anti-tumor immunity, however, the majority of the transferred tumor antigen-specific CD8^+^ T cells differentiated into CD39^+^CD69^+^ exhausted progenies, limiting its effects in repressing tumor growth. Contrarily, less attention has been addressed to the role of CD4^+^ T cells during tumorigenesis. Using a mouse model of metastatic melanoma, we found that transferring tumor-specific CD4^+^ T cells into recipients induces substantial regression of the established metastatic tumors. Notably, *in vitro* activated CD4^+^ T cells developed into cytotoxic CD4^-^ T cells *in vivo* and get exhausted gradually. The blockade of PD-L1 signaling resulted in an expansion of tumor specific CD4^+^ T cells, which could better control the established metastatic melanoma. Moreover, the tumor-specific memory CD4^+^ T cell can prevent mice from tumor metastasis, and the tumor-specific effector CD4^+^ T cells can also mitigate the established metastatic tumor. Overall, our findings suggest a novel mechanism of CD4^+^ T cells in curtailing tumor metastasis and confirm their therapeutic role in combination with PD-L1 blockade in cancer immunotherapy. Hence, a better understanding of cytotoxic CD4^-^ T cell-mediated tumor regression could provide an alternative choice for patients exhibiting suboptimal or no response to CD8^+^ T cell-based immunotherapies.

## Introduction

Metastasis is the primary cause of death in patients with tumors (1, 2). Of patients with sarcoma, 80% harbor metastases, as do 50% of those with colorectal cancer (3). Surgery, chemotherapy, and radiotherapy can reduce primary lesions; however, effective treatment for patients with distant metastasis remains a major challenge. Immunotherapy, including immune checkpoint blockade (ICB) and adoptive cell therapy, has been widely used for cancer treatment. Unfortunately, only about 30% of patients respond to ICB (immune checkpoint blockade) therapy by PD-1 blockade. Furthermore, clinical treatment often has to be ceased due to lethal side effects (4). Based on their direct role in tumor cell cytolysis, adoptive T cell therapy using tumor-specific T cells has long focused on cytotoxic CD8^+^ T cells, however, the transferred tumor-specific CD8^+^ T cells mostly differentiate into CD39^+^CD69^+^ exhausted cells, which are poorly maintained as tumor progresses (5), leading to limited success in tumor repression. Moreover, some reports have suggested that patients can respond to PD-1/PD-L1 blockade independently of CD8^+^ T cell responses (6, 7), indicating that other cell types, including CD4^+^ T cells, might contribute to the outcomes of this therapy.

Although CD4^+^ T cells are critical for promoting and sustaining CD8^+^ T cell responses during chronic viral infection (8), recent research has revealed more definite roles of CD4^+^ T in anti-tumor immunity. Adoptive transfer of Th9/Th17 cells induced *in vitro* can be well maintained *in vivo*, where they kill tumors by releasing Granzyme B (9, 10). Further, naïve tumor-specific CD4^+^ T cells can naturally differentiate into Th1 cytotoxic T cells *in vivo* and cause the regression of established tumors in hosts with lymphopenia (11), supporting the potential for the use of tumor-reactive CD4^+^ T cells in cancer immunotherapy. These findings have been validated in humans, where the adoptive transfer of large numbers of CD4^+^ T cells expanded from a tumor-specific CD4^+^ T-cell population resulted in complete response in patients with melanoma and cholangiocarcinoma (12, 13). In human bladder cancer, intra-tumoral cytotoxic CD4^+^ T lymphocytes, rather than canonical CD8^+^ T lymphocytes, mediate anti-cancer immunity (14); CD4^+^T cells could also exert their anti-tumor immunity by secretion of IFN-γ and TNF for recruitment of effector immune cells and the induction of tumor senescence (15, 16). However, the precise mechanisms leading to tumor regression remain elusive. In addition, whether antigen-specific memory CD4^+^ T cells can prevent tumor metastasis and respond to PD-1 signaling blockade therapy has not been fully elucidated, and the possibility that CD4^+^ T cells can become exhausted during tumor progression remains a matter of debate.

Here, We used T-cell receptor transgenic mice specific for CD4^+^ T cell epitopes LCMV-GP 66-77 (SMARTA mice) and OVA 323–339 (OT-II mice) as models to study tumor-specific CD4^+^ T cells during cancer immunotherapy. We found that the transferred tumor-specific CD4^+^ T cells efficiently mitigated melanoma lung and liver metastases, independent of CD8^+^ T, natural killer (NK), and macrophage cells. The majority of transferred tumor-specific CD4^+^ T cells differentiated into Th1 cells and acquired cytolytic function, accompanied by a gradual loss of the expression of CD4. Moreover, we demonstrated that tumor-specific CD4^+^ T cells also became exhausted during tumor progression and that PD-L1 administration caused a proliferative burst of the tumor-specific CD4^+^ T cells, which could eliminate lung metastasis. In addition, our data demonstrate that infection-induced antigen-specific effector CD4^+^ T cells can be used to treat tumor metastases, while antigen-specific memory CD4^+^ T cells can maintain protective immunity against tumor metastasis. Together, these results suggest that the anti-tumor effects of tumor-specific CD4^+^ T cells might have been underestimated. Hence, a better understanding of tumor-specific CD4^+^ T cells and regulation of their function in adoptive cell therapy may contribute to the development of effective cancer immunotherapies.

## Materials and Methods

### Mice

C57BL/6J, *Cd4* ^–/–^, *Cd8* ^–/–^, OT-II transgenic mice (specific for chicken ovalbumin-derived peptide OVA_323-339_ in the context of I-A^b^) and 45.1^+^ congenic mice were purchased from the Jackson Laboratory. SMARTA transgenic mice (specifically recognizing LCMV glycoprotein-derived peptide GP_66–77_ presented by I-A^b^) were kindly provided by Rafi Ahmed (Emory University). All mouse strains were on a C57BL/6J background and were housed and bred under specific-pathogen-free condition. Mouse experiments were performed following the guidelines of the Institutional Animal Care and Use Committees of the Army Medical University. Mice were infected/immunized at 6–10 weeks of age. The lymphocytic choriomeningitis virus (LCMV) Armstrong was gifted by Rafi Ahmed (Emory University) and propagated in our laboratory as previously described (17). Mice were intraperitoneally injected with 2 × 10^5^ plaque-forming units of Armstrong to establish an acute viral infection model. At least three animals from each group, matched for age and sex, were analyzed in each experiment.

### Cell Lines and Tumor Challenge

The tumor cell lines B16F10-OVA (referred to as B16-OVA hereinafter) and MC38-OVA (B16F10 murine melanoma cells or MC38 colon adenocarcinoma cells stably expressing chicken ovalbumin protein) were purchased from ATCC, and B16F10-GP (B-Luciferase B16F10 melanoma cells stably expressing LCMV glycoprotein, referred to as B16-GP hereinafter) was constructed by Beijing Biocytogen Co.Ltd, China. All the tumor cells were cultured in the D10 medium, comprising DMEM (Gibco, Cat. C11995500BT), 10% FBS (Gibco, Cat. 10270-106), 1% L-Glutamine (Solarbio Cat. G0200), and 1% penicillin/streptomycin (Gibco, Cat. 15070-063). For B16-GP melanoma cells, an additional 100U/ml puromycin (Sigma, Cat. 58-58-2) was supplemented. Mice were challenged with 5 × 10^5^ B16-GP or B16-OVA tumor cells intravenously (i.v.) for the lung metastasis model. The liver metastasis model was developed as previously reported (18). In brief, a total of 2 ×10^5^ B16-GP cells were injected into the spleen and 3 min after cell implantation, splenectomy was conducted. For *in situ* tumor model, mice were subcutaneously (s.c.) inoculated with 1 × 10^6^ B16-GP or MC38-OVA tumor cells. The subcutaneous tumors were measured every 2 days post cell transfer with a vernier caliper and the tumor volume was calculated according to the formula (length × width^2^)/2. Mice in metastatic models were sacrificed at the indicated time points with the numbers of metastatic foci calculated.

### Luciferase Reporter Assay

Mice were injected intraperitoneally with 200 µL of 2.5 mg/mL D-luciferin potassium salt(Perkin Elmer, Cat.122799), and 3 min afterwards, the bioluminescence images were taken using the IVIS imaging system.

### Lymphocyte Separation

Spleens were resected with sterilized scissors and crushed with the blunt part of a 1 mL syringe on Petri dishes containing 2 mL red blood cell lysis buffer. The resulting cell suspensions were filtered through a 70 μM filter into a 15 mL conical tube, centrifuged at 1800 rpm for 6 min at 4°C, and the supernatants were discarded. Cells were resuspended in 3 mL of R2 medium: RPMI-1640 (Sigma, Cat. RNBH7001) supplemented with 2% FBS. Lungs were first perfused with sterile PBS through the right ventricle to remove residual blood and subsequently cut into pieces and digested with type 2 collagenase (Sangon Biotech, Cat. A004174-0001) by shaking at 250 rpm for 1 h at 37°C. Then the samples were homogenized with the blunt part of a 1 mL syringe, filtered through a 70 μM filter into 50 mL conical tubes, and washed with R2 medium before centrifugation. The B16-GP tumor cells were further fractionated by centrifugation at 2000 rpm for 30 min at 22°C on a gradient comprising 44% and 67% Percoll solutions (GE, Cat. 17-0891-09); the T-cell fraction was recovered from the interface between the two layers.

### Cell Purification, *In Vitro* Culture and Adoptive Transfer

CD4^+^ T cells of SMARTA or OT-II mice were isolated by negative selection. Briefly, mice splenocytes were subjected to lineage depletion using biotin-conjugated antibodies (CD8, B220, CD11b, CD11c, Gr-1, TER119, CD44, CD25, and NK1.1; the clone, dilution, and provider of each antibody are listed in **Supplementary Table 1**), coupled with Beaver Beads Mag500 Streptavidin Matrix (Beaver, 22302). CD4^+^ T cells (purity > 90%) were then stimulated in the presence of plate-bound anti-CD3 (1μg/mL) and anti-CD28 (1μg/mL) for 48 h in complete R10 medium (RPMI-1640 supplemented with 10% FBS, 2 mM L-glutamine, 10 mM pyruvate [Sigma, Cat. S8761], 1% penicillin/streptomycin, 10 mM HEPES [Sigma, Cat. 83264], and 50 µmol β-mercaptoethanol [Procell, Cat. PB180633] containing IL-2 (20 ng/mL). Cells were removed from anti-CD3 and anti-CD28 48 h later and cultured in complete media containing 20 ng/mL IL-2 for another 7 days. About 12~24 hours prior to adoptive cell transfer, recipient mice were intraperitoneally injected with 200 mg/kg cyclophosphamide (CTX, Sigma) to create empty “space” within the lymphoid compartment for the adoptively transferred T cells by transiently depleting proliferating lymphocytes in mouse models. On Day 8 after tumor cell inoculation, a total of 2 × 10^6^ activated CD4^+^ T cells were adoptively transferred into tumor-bearing mice.

### *In Vivo* Treatments

For CD8^+^ T and NK cells depletion experiments, 200 μL PBS containing 50 µg of anti-CD8 (YTS-169.4, BioXcell, Cat. BE0117) or anti-NK (PK136, Biolegend, Cat. 108701) monoclonal antibodies or not was injected into tumor-bearing mice intraperitoneally (i.p.) 2 days before initiation of adoptive cell transfer and every subsequent 2 days for a total of five injections. For depletion of macrophages, mice were intranasally administrated with 50μL of Clodronate Liposomes (5 mg/ml; LIPOSOMA, Cat.CP-005-005) or PBS control on Day -2 of adoptive cell transfer and every 2 days post-transfer for a total of four doses. For ICB experiments, i.p. injections for 150 μg of anti-PD-L1 (RMP1-14, BioXCell) monoclonal antibody or PBS control were performed every 2 days after adoptive cell transfer, four doses in total. FTY720 treatment was given i.p. of 25 μg in PBS every 2 days from Day 1 to Day 7 post-cell transfer.

### *Ex Vivo* Killing Assay

B220^+^ cells (MHC-II-positive), as target cells, were collected from the spleen of C57BL/6J mice (CD45.1^+^CD45.2^+^) and purified by negative selection, subsequently labeled with CellTrace Violet (Life Technologies) at either 1 μM or 100 nM. Cells labeled with 1 μM Cell Tracer Violet were then pulsed with 1 μM GP66-77 peptide (recognized by SMARTA cells) for 1h at 37°C, and 100 nM-labeled cells with 1 μM GP33-41 peptide as control (not recognized by SMARTA cells). These cells were rinsed three times in RPMI-1640 containing 10% FBS. The Violet-high (1 μM) target cells were mixed with Violet low (100 nM) control cells at a ratio of 1:1, which were co-cultured with SMARTA cells (CD45.1^+^) sorted from the tumor-bearing mice at the effector to target ratio of 10:1 for 16 h in a cell incubator (37°C, 5%CO_2_). Killing efficiency was calculated as previously described (19): 100 − ([(% peptide pulsed in effector group/% un-pulsed in effector group)/(% peptide pulsed in control group/% un-pulsed in control group)] × 100).

### Flow Cytometry

Flow cytometric analysis was performed on a FACSCanto II or a FACSFortesa instrument (BD Biosciences). All the antibodies used for flow cytometry is listed in **Supplementary Table 1**. Surface staining was performed in PBS containing 2% bovine serum albumin or FBS (w/v). 0.5~1×10^6^ cells for each sample were stained with surface antibody cocktails for 30 min on ice. For the detection of transcription factors such as T-bet, PU.1, RORγt, and Foxp3, surface-stained cells were permeabilized, fixed and stained by using the Foxp3/Transcription Factor Staining Buffer Set (eBioscience, 00-5523) according to manufacturer’s instructions. For the detection of intracellular cytokine production, SMARTA cells were stimulated for 5 h at 37°C with 0.2 μg/mL of GP66–77 peptide or phorbol myristate acetate (PMA)/ionomycin in the presence of brefeldin A and DNase I (10 μg/ml). Following surface staining, cytokines including IFN-γ, TNF-α, IL-2, granzyme B, and granzyme A were stained with a Cytofix/Cytoperm Fixation/Permeabilization Kit (554714, BD Biosciences) according to the manufacturer’s instructions. Flow cytometry data were analyzed with FlowJo software (Tree Star).

### Statistical Analysis

Statistical analysis was conducted with Prism 7 software (GraphPad). Two-tailed paired Student’s t-test, two-tailed unpaired Student’s t-test, or one-way ANOVA with Newman–Keuls’s test were used to calculate P-values. Two-way ANOVA with a Turkey *post hoc* test was performed for comparing tumor growth curves at different time points and the log-rank (Mantel–Cox) test for comparing mouse survival curves. *p* values < 0.05 were considered significant (*: *p* < 0.05; **: *p* < 0.01; ***: *p* < 0.001; ****: *p* < 0.0001); *p* values > 0.05: non-significant (ns).

## Results

### Tumor-Specific CD4^+^ T Cells Mitigate Metastatic Melanoma Tumor Progression in Mice

**Figure 1:**
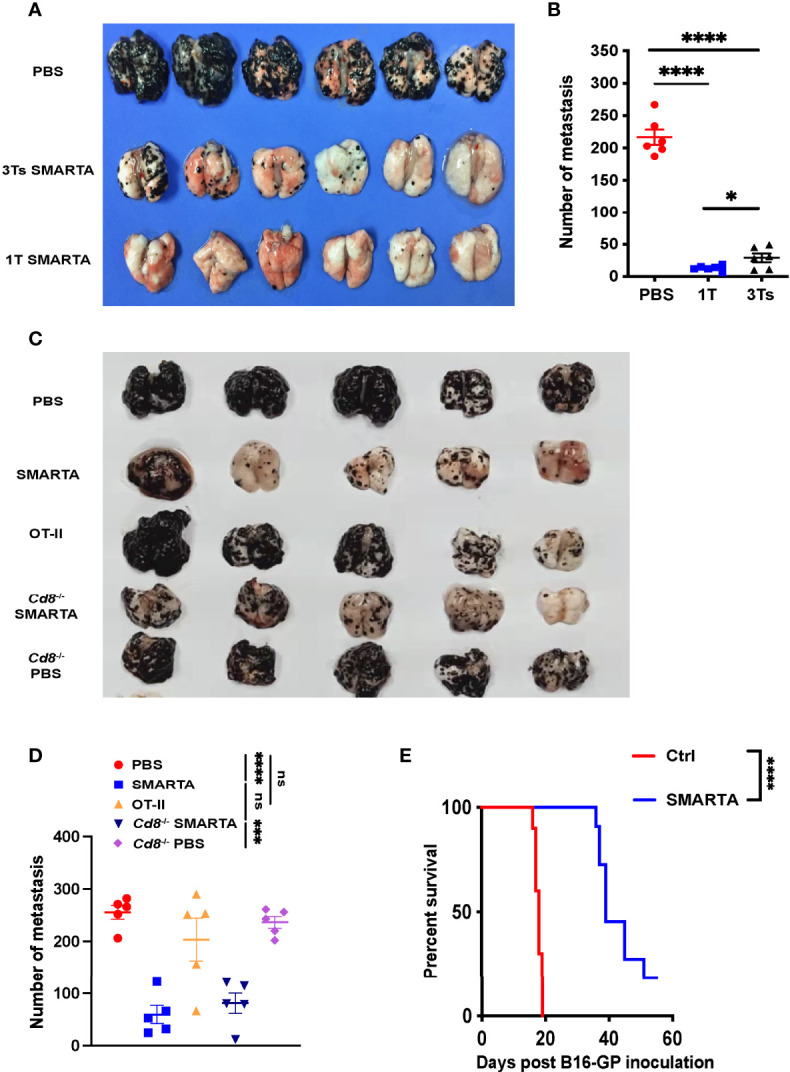
Transfer of tumor-specific CD4^+^ T cells potently restricts lung metastasis from melanoma. 0.5 × 10^6^ B16-GP cells were injected intravenously into C57BL/6J mice (CD45.2^+^) through tail vein to develop the lung metastasis. On Day 7, tumor-bearing mice were administered with CTX (200mg/kg) intraperitoneally and transferred with 2 × 10^6^ CD45.1^+^activated tumor-specific CD4^+^ T cells or PBS (control) intravenously 12 hours later (Day 8). **(A, B)** Image of lung samples harvested from metastasis model in which C57BL/6J mice (n=6/group) were treated with PBS or 2 × 10^6^ SMARTA cells in total (once or in three divided doses for 3 consecutive days) and sacrificed seven days post-transfer **(A)**, with the numbers of metastatic foci calculated **(B)**. **(C, D)** Image of lung samples harvested from the lung metastasis model in which the C57BL/6J mice (n=5/group) with established B16-GP lung metastasis were treated with PBS, activated SMARTA or OT-II cells, and B16-GP bearing *Cd8* ^-/-^ mice (n=5/group) were treated with either activated SMARTA cells or PBS **(C)**. Mice were sacrificed on Day 18 post transfer. The statistical analysis of the numbers of metastatic foci in the lung tissues **(D)**. **(E)** Survival curve of tumor-bearing mice (n=10/group) treated with activated SMARTA cells or control PBS. Statistical differences are calculated by one-way ANOVA (**B**, **D**, number of metastasis) and Log-rank test (**E**, survival curve). ns, not significant, *p < 0.05, ***p < 0.001, ****p < 0.0001. Data are presented as mean ± SEM.

To determine whether the adoptive transfer of *in vitro* activated tumor-specific CD4^+^ T cells could mitigate established metastatic melanoma tumors *in vivo*, we used T-cell receptor transgenic CD4^+^ T cells purified from SMARTA (CD45.1^+^ LCMV GP66–77 I-A^b^-specific) or OT-II (CD45.1^+^ chicken ovalbumin 323–339 I-A^b^-specific) transgenic mice. The purified CD4^+^ T cells from the spleen and superficial lymph node were activated with anti-CD3 and CD28 antibodies and sustained by IL-2 supplementation as described. C57BL/6 mice were administered 5 × 10^5^ melanoma B16-GP or 5 × 10^5^ B16-OVA i.v. On Day 8, when lung metastasis was established as evidenced by luciferase reporter assay (**Supplementary Figure 1A**), 2 × 10^6^ activated SMARTA CD4^+^ T cells in total (one dose or divided into three doses for successive 3 days) were transferred into B16-GP tumor-bearing mice i.v. by tail vein injection. Seven days after CD4^+^ T-cell transfer, mice were sacrificed and the numbers of metastasis foci were determined. There were significantly fewer metastatic foci in the transferred groups than that in the control groups (**Figures 1A, B**), while the efficacy was most significant in the one dose transferred group. Thus, one-dose transfer strategy was chosen for the following experiment. Transfer of 2 × 10^6^ activated OT-II cells into B16-OVA tumor-bearing mice also resulted in substantial metastasis remission (**Supplementary Figures 1B, C**).

Next, to determine whether tumor restriction was antigen-dependent, activated OT-II cells were transferred into B16-GP bearing mice on Day 8 after tumor inoculation, the transferred activated OT-II T cells did not show any effect on B16-GP tumor metastasis (**Figures 1C, D**), indicating that anti-tumor activity was mediated by SMARTA cells after CD4^+^ T-cell transfer, and not by unrelated CD4^+^ T cells. We conclude that the protection mediated by CD4^+^ T cells is antigen-dependent.

Without treatment, all mice died around 20 days after injection of tumor cells; however, transfer of activated tumor-specific CD4^+^ T cells significantly prolonged mouse survival (**Figure 1E** and **Supplementary Figure 1C** right panel). To further elucidate whether the anti-tumor efficacy was specific to lung metastasis, B16-GP liver metastasis was induced and activated SMARTA cells were transferred. The transferred tumor-specific CD4^+^ T cells also significantly reduced liver metastasis (**Supplementary Figures 1D, E**), demonstrating that the effect of the tumor-specific CD4^+^ T cells on metastasis was a general phenomenon.

### Tumor-Specific CD4^+^ T Cell-Mediated Tumor Rejection Is Independent of Endogenous T Cells, NK Cells, and Macrophages

CD4^+^ T cells can interact with many other cell types to execute their functions. For example, they have critical roles in enhancing CD8^+^ T-cell responses (8, 20) and coordinate with NK cells and macrophages to eradicate tumors (21–25). After activated SMARTA cell transfer, the absolute number of endogenous tumor-reactive CD4^+^and CD8^+^ T cells (as determined by CD44^+^PD-1^+^ (26–28)) increased dramatically (**Supplementary Figures 2A, B**). So did the absolute number of macrophages and NK cells (**Supplementary Figure 2C**). To determine the role of these host cells played in the control of metastasis following activated tumor-specific CD4^+^T cell transfer, we used *Cd4* ^–/–^ mice, *Cd8* ^–/–^ mice as recipient mice and found that CD4^+^ T-cell adoptive transfer also significantly reduced metastatic foci in the lung of these mice (**Figures 1C, D** and **Supplementary Figure 3A**). In an alternative way, depletion antibodies were used and the depletion of CD8^+^ T, NK cells, and macrophages was confirmed by flow cytometry (**Supplementary Figure 3B**). However, activated antigen-specific CD4^+^ T-cell adoptive transfer significantly reduced metastatic foci in the lung, irrespective of endogenous cell depletion, indicating that tumor control is independent of endogenous CD4^+^ T, CD8^+^ T, NK cells, and macrophages (**Figures 1C, D** and **Supplementary Figure 3**).

### Tumor-Specific CD4^+^ T Cells Differentiate Into Cytotoxic CD4^–^ T Cells, Which Is Critical for Tumor Rejection

**Figure 2:**
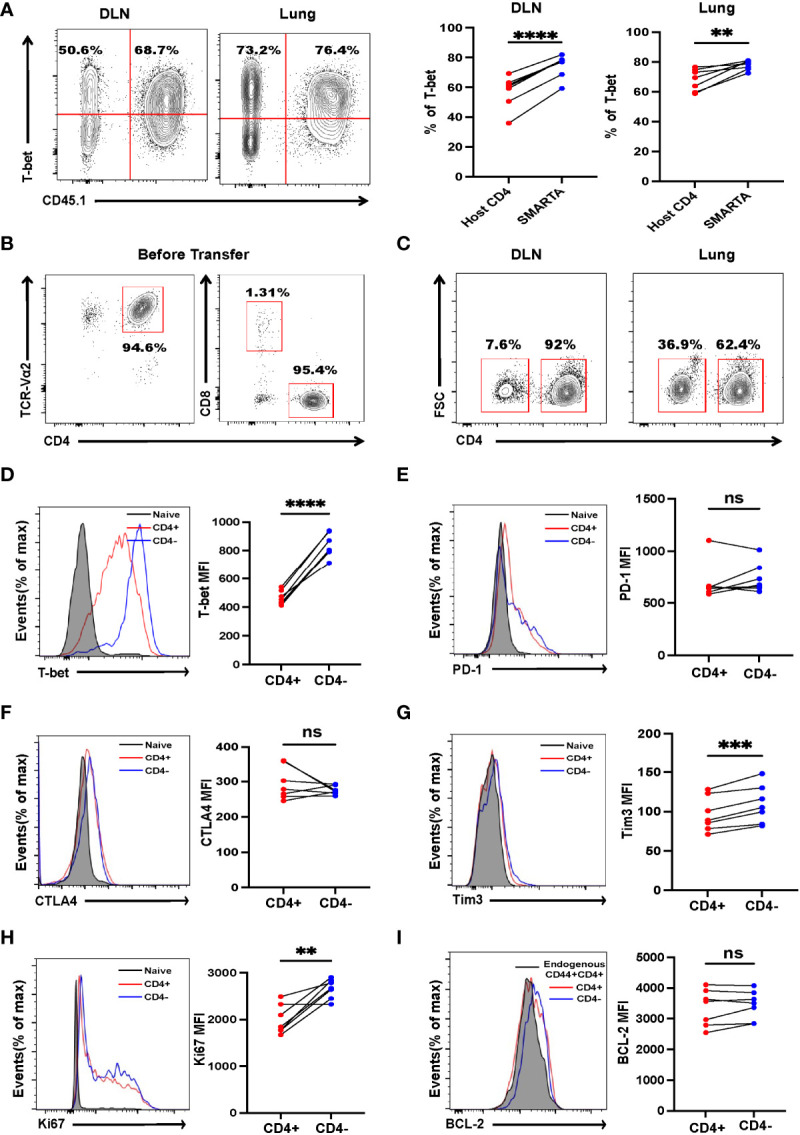
Tumor-specific CD4^+^ T cells differentiate into Th1 and CD4^-^ T cells. B16-GP tumor-bearing C57BL/6J mice were adoptively transferred with 2 × 10^6^ activated SMARTA cells on Day 8 after tumor inoculation and sacrificed seven days later. **(A)** Left panel: representative flow cytometry plots of T-bet expression in CD4^+^ T cells in draining lymph node (DLN) and lung tissue (Lung). Cells are gated on live CD44^+^CD4^+^ T cells. The numbers are percentages of cells accounting for CD45.1^-^(left quadrant) and CD45.1^+^ (right quadrant) populations. Right panel: the statistical analysis of percentages of T-bet positive cells as shown in the left panel (n=7/group). **(B)** The purity of the activated SMARTA cell before transfer. **(C)** Representative flow cytometry plots of CD4^+^ and CD4^–^ SMARTA cells in the DLN and lung and cells are gated on live CD45.1^+^ cells. Numbers are frequencies of indicated populations (n=7/group). **(D-I)** Flow cytometry analyses of SMARTA cells isolated from the lung comparing the expression level of T-bet, PD-1, CTLA4, Tim3, Ki67 and BCL-2 between CD4^+^ and CD4^-^ SMARTA cells. The mean fluorescent intensities (MFIs) are summarized beside. Cells are gated on live CD45.1^+^ cells (n=7/group). Statistical differences are calculated by paired student’s *t* test. ns, not significant, **p <0.01, ***p < 0.001, ****p < 0.0001.

**Figure 3:**
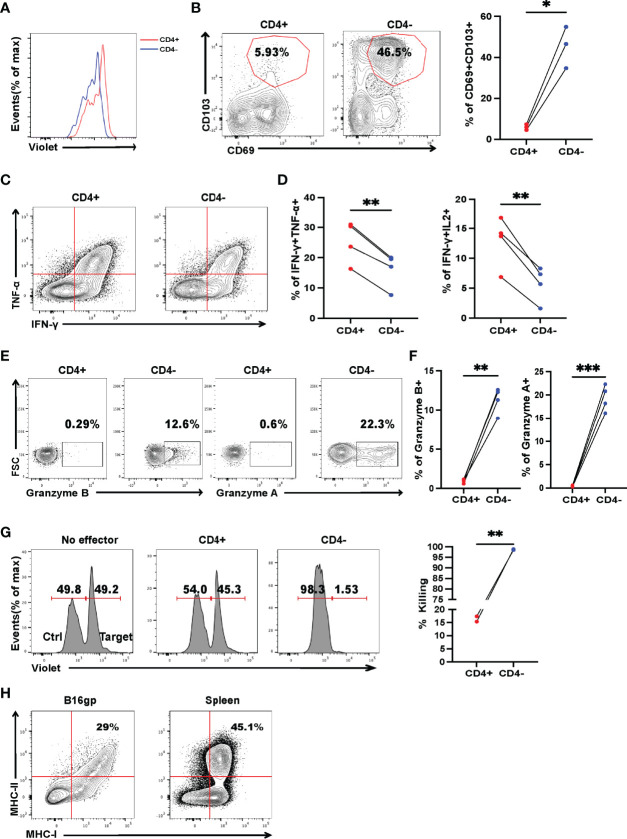
Cytotoxic CD4^–^ T cells, differentiated from CD4^+^ T cells, are critical for controlling established tumor metastasis. Tumor-bearing mice were treated with 2 × 10^6^ activated SMARTA cells eight days after tumor inoculation and sacrificed on Day 7 or Day 15 post-transfer. **(A)** Flow cytometry analysis of the dilution pattern of the cell proliferation dye on CD4^+^ and CD4^–^ SMARTA cells which were sorted from the metastatic lung tissue on Day 7 post cell transfer and labeled with CellTrace Violet, followed by *in vitro* stimulation of plate-bound anti-CD3(1ug/ml) and soluble anti-CD28(1ug/ml) for 72 h Cells are gated on live CD45.1^+^ cells. **(B)** Representative flow cytometry plots of CD69 and CD103 expression in CD4^+^ and CD4^–^ SMARTA cells on Day 15 post cell transfer. The frequencies of CD69^+^CD103^+^ cells in each population are summarized beside. (n=3/group). **(C, D)** Representative FACS data of TNF-α and IFN-γ production of CD4^+^ and CD4^–^ SMARTA cells after *in vitro* GP66-77 re-stimulation on Day 7 post-SMARTA transfer. Frequencies of IFN-γ^+^ TNF-α^+^ and IFN-γ^+^ IL-2^+^ cells in the indicated populations are summarized in **(D)**. (n=4/group). **(E, F)** Representative FACS data of Granzyme A and Granzyme B production of CD4^+^ and CD4^–^ SMARTA cells after GP66–77 re-stimulation on Day 15 post cell transfer. Frequencies of Granzyme B^+^ and Granzyme A^+^ cells in the indicated populations are summarized in **(F)**. (n=4/group). **(G)** Flow cytometry analyzing the killing capacity of CD4^+^ and CD4^–^ SMARTA cells, in which Violet-high B220^+^ cells loaded with MHC class II-restricted peptide GP66–77 (recognized by SMARTA cells) were target cells, whereas Violet-low cells labeled with GP33–41 as control; the effector: target ratio is 10:1. The percentage of killing by the populations is summarized beside. **(H)** Flow cytometry plots of MHC-I and MHC-II expression in B16-GP cells harvested from metastatic lung or in splenocytes of C57BL/6J mouse (positive control). Statistical differences are calculated by paired student’s *t* test. *p <0.05, **p <0.01, ***p < 0.001.

CD4^+^ T cells can differentiate into Th1, Th2, Th9, and Th17 lineages to eradicate established tumors (9, 12, 29, 30); however, little is known about *in vivo* differentiation of adoptive transferred activated CD4^+^ T cells in the context of metastasis. Mice were sacrificed 7 days after transfer, lymphocytes from the draining lymph nodes and lungs were analyzed by flow cytometry. Despite most of the activated SMARTA cells exhibit an unskewed phonotype before transfer (**Supplementary Figure 4**), the majority of SMARTA cells from the draining lymph node (DLN) and lung were found to differentiate *via* the Th1 program, as evidenced by high expression of T-bet (**Figure 2A**), but not CXCR5, FOXP3, PU.1 or RORγt (**Supplementary Figures 5A–D**), indicating that Th1 cells are critical for tumor control. Surprisingly, although most of the activated SMARTA cells before transfer were CD4^+^T cells (**Figure 2B**), we did find that a large fraction of donor CD4^+^ T cells became CD4^–^ *in vivo*, which was more evident in the lung than that of the DLN (**Figure 2C** and **Supplementary Figure 6A**). This finding reminded us whether the contamination of other cells could account for the appearance of CD4^-^ T cells. So, we checked the expression of TCRβ and other lineage markers including CD8, B220, CD19, NK1.1, F4/80, CD11b, CD11c and TER-119, and confirmed that these SMARTA cells are T cells with little contamination (**Supplementary Figure 6B**). Previous studies showed that CD4 molecules could be internalized *in vitro* or converted into CD8^+^ T cells (31, 32); however, CD8 expression was almost absent in these SMARTA cells, and intracellular CD4 and CD8 staining did not alter the proportion of CD4^+^ T cells (**Supplementary Figure 6C**). Further, the identified CD4^–^ T cells expressed comparable levels of the inhibitory markers, PD-1, CTLA4 but higher Tim3 and T-bet to CD4^+^ T cells (**Figures 2D–G**), and they expressed higher levels of Ki67 but the comparable level of BCL-2 (**Figures 2H, I**). After stimulation *in vitro* with anti-CD3 and CD28, the CD4^-^ cells showed a more robust proliferation response, relative to their CD4^+^ T-cell counterparts (**Figure 3A**). Moreover, the expression levels of CD69 and CD103 in CD4^–^ T cells were significantly higher than those of CD4^+^ T cells, indicating tissue residency (**Figure 3B**). After stimulation with GP66–77 peptide, the percentage of IFN-γ^+^TNF-α^+^ and IFN-γ^+^IL-2^+^ cells were significantly lower (**Figures 3C, D**); however, they secreted much larger amounts of Granzyme A and B (**Figures 3E, F**), indicating that these CD4^-^ cells may have cytolytic function. Indeed, *in vitro* killing assays showed that CD4^–^ SMARTA cells exhibited a superior killing capacity to CD4^+^ SMARTA cells (**Figure 3G**), consistent with the finding that MHC-II was expressed on B16-GP cells *in vivo* (**Figure 3H**). The percentages of CD4^–^ cells were consistently higher in the lung than those in DLN (**Supplementary Figure 6A**), indicating that antigen load may contribute to CD4^–^ cell induction. To directly test this hypothesis, purified SMARTA cells were cultured *in vitro* with different concentrations (1µg/mL and 10 µg/mL) of plate-coated anti-CD3 antibody, supplemented with anti-CD28 and IL-2. Almost all SMARTA cells in the 1 µg/mL group maintained their CD4 expression 8 days after culture, while approximately 15% of SMARTA cells in the 10 µg/mL group lost CD4 expression, mimicking the phenotype observed *in vivo* (**Supplementary Figure 6D**).

### Tumor-Specific CD4^+^ T Cells Have Altered Effector Functions During Tumor Progression

**Figure 4:**
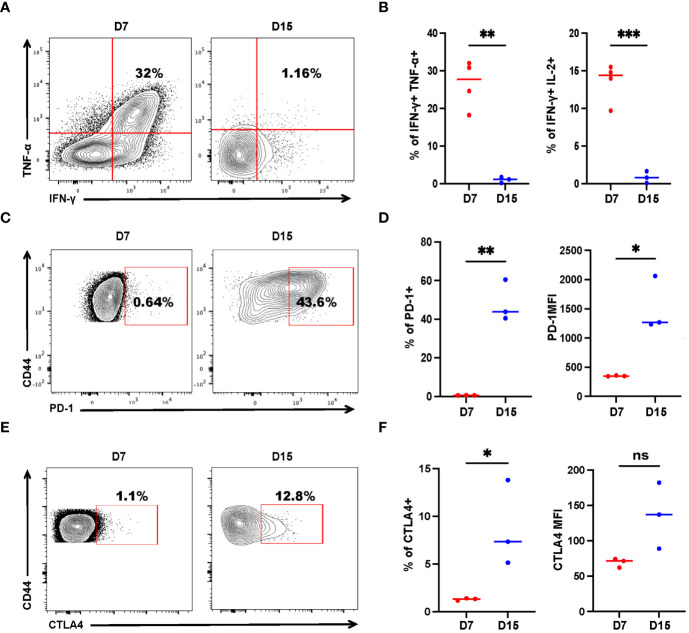
Tumor-specific CD4^+^ T cells get exhausted during tumor progression. Tumor-bearing mice were treated with 2 × 10^6^ activated SMARTA cells eight days after tumor inoculation and sacrificed on Day 7 or Day 15 post-transfer. Cells are gated on live CD4^+^CD44^+^CD45.1^+^ cells. **(A, B)** Representative flow cytometry plots of intracellular cytokine staining of CD4^+^ SMARTA cells on Day 7 (n=4) and Day 15 (n=3) post-SMARTA transfer **(A)**. The frequencies of IFN-γ^+^ TNF-α^+^ and IFN-γ^+^ IL-2^+^ cells of CD4^+^SMARTA cells **(B)**. **(C, D)** In a separate experiment, representative flow cytometry plots of PD-1 expression in CD4^+^ SMARTA cells on Day 7 and Day 15 (n=3/group) post-transfer **(C)**. The frequency of PD-1^+^ SMARTA cells and MFI of PD-1 **(D)**. **(E, F)** Representative flow cytometry plots of CTLA4 expression in CD4^+^ SMARTA cells on Day 7 and Day 15 (n=3/group) post SMARTA transfer **(E)**. The frequency of CTLA4^+^ SMARTA cells and MFI of CTLA4 **(F)**. Statistical differences are calculated by unpaired *t*-test. ns, not significant, *p < 0.05, **p < 0.01, ***p < 0.001.

While CD8^+^ T-cell exhaustion is relatively well described, much less is known about the exhaustion of CD4^+^ T cells in cancer. Adoptive transfer of tumor-specific CD4^+^ T cells could mitigate metastasis. However, few of the mice were tumor-free, indicating that the tumor-specific CD4^+^ T cells may have been exhausted, akin to CD8^+^ T cells, as antigens persisted. To study tumor-specific CD4^+^ T-cell dysfunction, we measured the expression of exhaustion markers, as well as IFN-γ, TNF-α, and IL-2 production of recovered SMARTA cells. On Day 7 post transfer, a sizable fraction (one-third) of donor cells concurrently produced multiple proinflammatory cytokines (IFN-γ, TNF-α) upon re-stimulation with the cognate peptide (**Figure 4A**), resembling the phenotype of so-called ‘polyfunctional effector cells’ which are more effective in controlling viral infections and tumor growth (33, 34). However, cytokine production was severely compromised on Day 15 (**Figures 4A, B**). Furthermore, SMARTA cells showed low expression of PD-1 and CTLA4 on Day 7, but on Day 15, the percentage of PD-1 and CTLA4 positive cells were higher than that of the cells on Day 7 and the mean fluorescence intensity of PD-1 was also upregulated (**Figures 4C–F**), indicating that CD4^+^ T-cell subsets also exhibit altered phenotypes, including features of exhaustion, during cancer progression.

### Tumor-Specific CD4^+^ T Cells Have With PD-L1 Blockade

**Figure 5:**
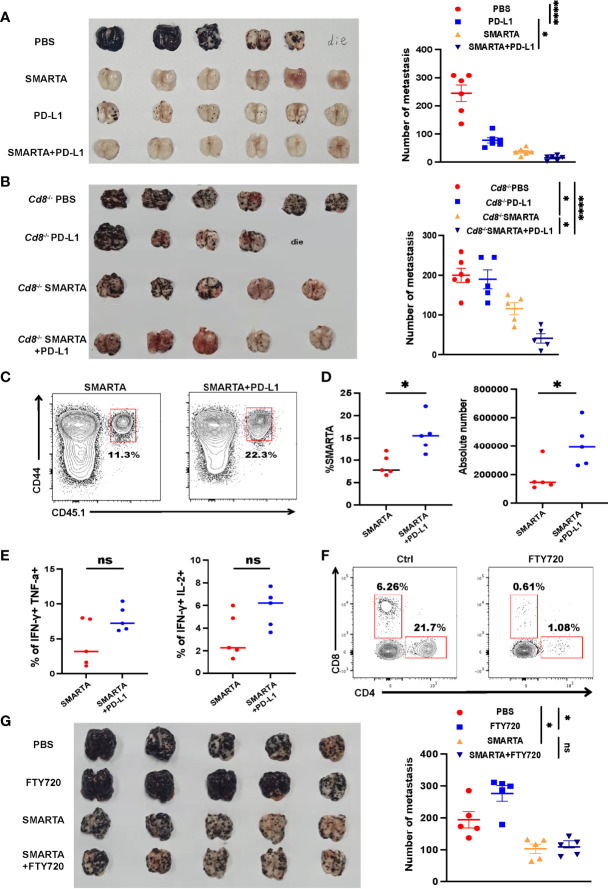
Tumor-specific CD4^+^ T cells have a synergistic therapeutic effect with PD-L1 blockade. **(A)** Representative image of lung samples harvested from tumor-bearing C57BL/6J mice (n=6/group) which were treated with 2 × 10^6^ activated SMARTA cells or PD-L1 blockade therapy alone or the two combined and sacrificed on Day 7 post transfer (endpoint). In PBS-treated group, a mouse died before the endpoint and metastatic foci on the mouse lung was calculated as the same value as the most severe one (similarly hereinafter). The numbers of the metastatic foci are summarized beside. **(B)** Representative image of lung samples harvested from tumor-bearing *Cd8* ^–/–^ mice which were treated with PBS (n=6) or PD-L1 monoclonal antibody (n=5) or SMARTA cells alone (n=5) or in combination (n=5) on Day 8 post tumor inoculation and were sacrificed on Day 15. In PD-L1 blockade-treated group, a mouse died before the endpoint. The statistical analysis of the metastatic foci is shown beside. **(C–E)** Flow cytometry analysis of transferred CD45.1^+^ CD44^+^ SMARTA cells in *Cd8* ^–/–^ mice with or without PD-L1 blockade therapy. The frequencies and absolute numbers of SMARTA cells **(D)** and the statistical analysis of cytokine production of SMARTA cells **(E)** in the two groups. **(F, G)** Tumor-bearing C57BL/6J mice (n=5/group) were transferred with SMARTA cells or PBS on Day 8 post tumor challenge, followed by three doses of FTY720 (25 µg intraperitoneal injection) or PBS treatment from Day 9 to Day 15. Representative flow cytometry plots of CD4^+^ and CD8^+^ T cells in the metastatic lung tissue after FTY-720 treatment analyzing the blocking efficacy of FTY-720 **(F)**. The image of lung tissue harvested from mice of indicated groups with the statistical analysis of the metastatic foci shown beside **(G)**. Statistical differences are calculated by one-way ANOVA (A, B, and G, number of metastasis), unpaired *t* test (D, E). ns, not significant, *p <0.05, ****p < 0.0001.

Tumor-specific CD4^+^ T cells express high levels of PD-1 after transferred into tumor-bearing mice; however, it remains to be determined whether CD4^+^ T-cell adoptive transfer has synergistic effect with ICB. Consistent with published data, anti-PD-L1 alone significantly reduced the mortality and repressed tumor growth, with results comparable to those in the CD4^+^ T-cell adoptive transfer group (**Figure 5A**). Although CD4^+^ T-cell adoptive transfer alone had considerable therapeutic efficacy, the combination of CD4^+^ T-cell adoptive transfer and anti-PD-L1 exhibited additional effects in delaying tumor growth (**Figure 5A**).

Next, to exclude the role of CD4^+^ T-cell help for CD8^+^ T-cell responses in the context of PD-L1 treatment, *Cd8* ^–/–^ mice were used as recipients. Anti PD-L1 treatment did not show improvement of the tumor control, SMATA cell adoptive transfer also limited the metastatic foci in CD8^-/-^ mice, however, the combination of CD4^+^ T-cell adoptive transfer and PD-L1 treatment again resulted in the best efficacy (**Figure 5B**). The percentage and the absolute number of SMARTA cells increased dramatically after PD-L1 blockade (**Figures 5C, D**); however, cytokine production seems to be increased but does not get statistical significance (**Figure 5E**). Overall, the results of this experiment indicate that adoptive CD4^+^ T-cell transfer could be a potential therapeutic approach for preventing metastasis in combination with ICB.

Lymph nodes have been recently recognized as critical contributors to cancer immunotherapy (35, 36). To evaluate whether lymph nodes are indispensable for adoptive CD4^+^ T-cell transfer therapy, FTY720 was administered to block lymphocytes migration from the lymph nodes to the periphery (**Figure 5F**). FTY720 blockade indeed aggravated lung metastasis in the control group, indicating that the lymph nodes played a critical role in controlling lung metastasis. However, FTY720 blockade did not mitigate CD4^+^ T-cell adoptive transfer efficacy (**Figure 5G**), consistent with the fact that these cells were activated *in vitro* and also in line with their tissue residency properties (**Figure 3B**).

### Antigen-Specific Effector CD4^+^ T Cells Can Control Lung Metastasis, While Memory CD4^+^ T Cells Can Prevent Lung Metastasis

**Figure 6:**
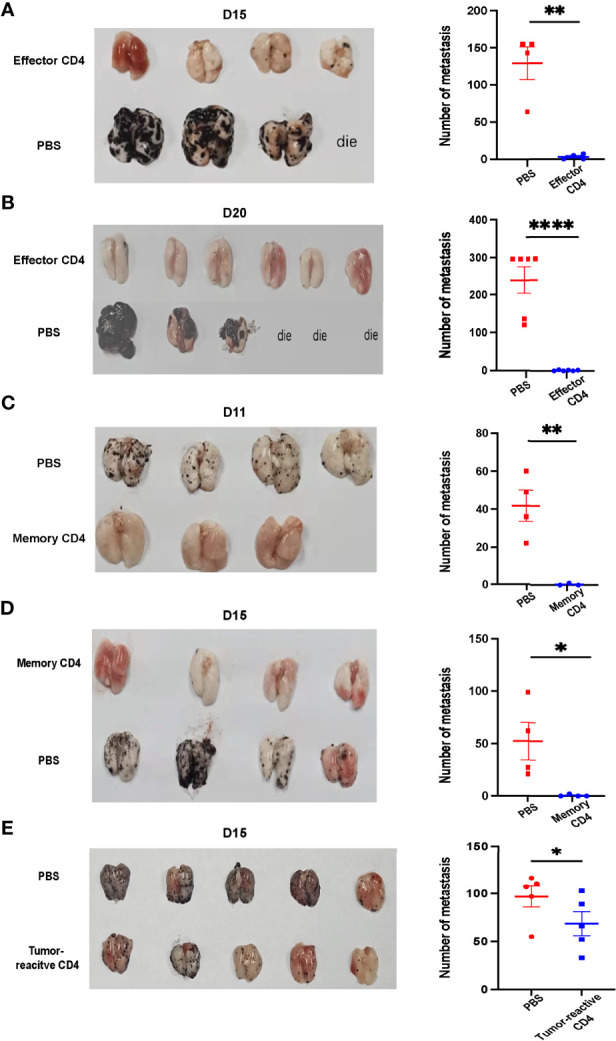
Antigen-specific effector CD4^+^ T cells can control lung metastasis while memory CD4^+^ T cells prevent mice from lung metastasis. **(A, B)** Image of lung samples of metastatic tumor models in which mice were inoculated with 5×10^5^ B16-GP cells intravenously and 8 Days later transferred with 1×10^6^ effector CD4^+^ T cells isolated from LCMV-Armstrong-infected mice. Tumor bearing mice were sacrificed on Day 15 **(A)** or Day 20 **(B)** after B16-GP inoculation. (n=4/group on Day 15, n=6/group on Day 20). Before Day 15, a mouse died and three mice died before Day 20. The statistical analyses of the metastatic foci are shown beside. **(C, D)** Image of lung samples of metastatic tumor models in which naïve mice were transferred with 1×10^5^ memory CD4^+^ T cells or PBS and then challenged with 5×10^5^ B16-GP. Mice were sacrificed on Day 11 **(C)** and Day 15 **(D)** post tumor challenge. On Day 11, n=4 in PBS group, n=3 in memory CD4 group, on Day 15, n=4/group. The statistical analyses of the metastatic foci are shown beside. **(E)** CD4^+^CD25^-^GITR^-^ T cells were separated from the lung tissue and the draining lymph nodes of B16-GP tumor-bearing mice eight days after tumor inoculation and 1.5×10^6^ PD-1^+^ CD44^+^CD25^-^CD4^+^T cells (tumor-reactive) or PBS were transferred into recipient mice (n=4/group) challenged with B16-GP four days before. The tumor-bearing recipient mice were sacrificed on Day 15 post tumor inoculation and the image of lung samples harvested from the mice and the statistical analysis of the metastatic foci is shown. Statistical differences are calculated by unpaired *t* test. *p < 0.05, **p < 0.01, ****p < 0.0001. Data are presented as mean ± SEM.

To further explore the role of antigen-specific CD4^+^T cells in the control or prevention of lung metastasis, 2,000 naïve SMARTA CD4^+^ T cells were adoptively transferred into congenic mice, followed by LCMV Armstrong infection. Eight days post-infection, CD4^+^ T cells were immunomagnetically enriched by negative selection. CD4^+^ T cells (purity > 91%) were subsequently transferred into pre-established (Day 8) B16-GP lung metastasis-bearing mice (1×10_6_ SMARTA cells/mouse). Numbers of metastatic foci were strikingly lower in the adoptively-transferred group than those in the control group on both Day 15 and Day 20 post tumor implantation (**Figures 6A, B**). Next, on Day 60 after LCMV Armstrong infection, when CD4 memory is well established, 1 × 10^5^ SMARTA memory CD4^+^ T cells were purified and transferred into naïve mice, which were then challenged with B16-GP. The transferred memory CD4^+^ T cells significantly protect the mice from lung metastasis relative to controls both on Day 11 and Day 15 after tumor inoculation (**Figures 6C, D**). To direct test whether endogenous tumor-reactive CD4^+^ T cells could control tumor metastasis, 1.5×10^6^ CD4^+^CD25^-^CD44^+^PD-1^+^T cells isolated from tumor-bearing mice (Day 8) or PBS were transferred into tumor-bearing recipient mice on Day 4 post tumor inoculation. The results showed that the endogenous tumor-reactive CD4^+^T cell only provided limited role in relieving lung metastasis (**Figure 6E**).

Next, we investigated whether activated tumor-specific CD4^+^ T cells could control solid tumors. Activated SMARTA cells (2 × 10^6^) were transferred into B16-GP tumor-bearing mice 8 days post-tumor implantation, while 2 × 10^6^ activated OT-II cells were transferred into MC38-OVA tumor-bearing mice 8 days after tumor implantation. Neither the activated SMARTA nor OT-II showed any effect on the solid tumor control (**Supplementary Figures 7A, B**). To further investigate the potential mechanisms beneath the different effects of activated SMARTA cells between metastatic tumor and solid tumor, activated SMARTA cells were transferred into one recipient which suffered from both solid and lung metastatic B16-GP tumor. We noticed that the absolute number of the activated SMARTA cells was significantly lower in the solid tumor than that in the metastatic tumor, while the percentages of Tim3, PD-1 positive cells were significant higher within solid tumor (**Supplementary Figures 7C, D**). The percentage and absolute number of IFN-γ^+^TNF-α^+^ and IFN-γ^+^IL-2^+^SMARTA cells were decreased dramatically in solid tumor than that in the lung metastasis (**Supplementary Figures 7E, F**). This data reminds us that antigen-specific CD4^+^ T cells response may be differentially regulated between these two tumor models, which needs further investigation.

## Discussion

Certain clinical outcomes have been achieved using ex vivo generated T cells in adoptive immunotherapy for metastatic melanoma (37–39) and tumor-reactive CD8^+^ T cells were infused into patients in most studies. CD8^+^ T cells are generally regarded as the cardinal immune cell type for controlling tumors due to their potent cytotoxicity, however, some tumor cells avoid elimination by downregulation of MHC class I expression (40). By contrast, the role of CD4^+^ T cells, particularly in the context of tumor metastasis, requires further delineation. In the present study, by activating tumor-reactive CD4^+^ T cells *in vitro*, we demonstrated that tumor-specific CD4^+^ T cells could delay metastatic foci formation, informing a potential strategy for future clinical treatment, particularly for patients who develop metastatic disease and for the patients who are resistance to CD8^+^ T cells because of the MHC class I loss (41, 42). Here, by using *Cd4* ^–/–^ and *Cd8* ^–/–^ recipient mice, we demonstrated that the adoptively transferred tumor antigen-specific CD4^+^ T cells can still exert potent anti-tumor effects even in the absence of endogenous CD4^+^/CD8^+^ T cells, which were further confirmed with the treatment of CD8^+^/CD4^+^ T depletion antibodies. Besides, we noticed that macrophages might play a certain role in the control of tumor, as evidenced by slightly increased metastatic foci in anti-macrophage group compared with the PBS group, which is consistent with published data reporting that tumor-specific CD4^+^ T cells can eliminate tumors *via* induction of macrophage cytotoxicity (24, 25). However, activated tumor-specific CD4^+^ T cells can restrain the metastatic foci, no matter macrophages are present or not, indicating that activated CD4^+^ T cells play a dominant role in tumor control in this model. We found that most transferred tumor-specific CD4^+^ T cells converted to Th1 cells and some of them gradually lost CD4 expression and differentiate into CD4^–^ T cells, which is consistent with previous report that upon chronic antigen exposure, helper T cells could down-regulate CD4 expression (43). We excluded that this population arose from other cells for the following reasons: first, these cells are all originated from SMARTA cells which have been enriched with the purity of the CD4^+^T cells > 90%; second, the majority of CD4^–^ T cells were negative for expression of other linage markers; third, they exhibited MHC-II-restricted killing activity, and could kill GP66–77-pulsed cells, without altering the survival of GP33–41-pulsed cells; and fourth, they showed potent cytokine production after GP66–77 peptide stimulation. By stimulation with GP66-77, the percentage of IFN-γ^+^TNF-α^+^ cells was significantly lower, while Granzyme B production was enhanced, reminiscent of terminally exhausted CD8^+^ T cells, although immune checkpoint markers PD-1, CTLA4 were not significantly altered and even exhibited higher proliferation. Our identification of a CD4^–^ CTL population may provide a platform for further investigations in humans of how and why CD4 expression is down-regulated, and thus provide insights into approaches with the potential to generate durable and effective CD4^–^ CTL immunity.

Exhausted CD8^+^ T cells are well described as exhibiting increased expression of inhibitory markers, commonly referred to as immune checkpoints, and a progressive and hierarchical loss of cytokine production. Although exhaustion of CD8^+^ T cells induced by cancer has been well-characterized and identified as a therapeutic target, whether CD4^+^ T-cell exhaustion occurs and its role in cancer has not been extensively investigated. Our data demonstrate that CD4^+^ T cells also gradually upregulate inhibitory markers and have functional deficits in cytokine production, providing evidence for CD4^+^ T-cell exhaustion. However, as with CD8^+^ T-cell exhaustion, a number of coinhibitory receptors may also serve to denote T-cell activation (44). Currently, it has been confirmed that CD4^+^ T cells exhibited a skewed differentiation toward follicular helper T cells during chronic viral infection, which has been induced by type I interferon (45). Further research is required to determine whether exhausted CD4^+^ T cells exhibit other features that mirror those of the exhausted CD8^+^ T-cell compartment: for example, loss of antigen-independent homeostatic proliferation capacity and acquisition of unique epigenetic features (46, 47). Furthermore, it will be interesting to determine whether CD4^+^ T-cell exhaustion evolves in a similar stage-dependent manner to that of CD8^+^ T cells.

Reinvigoration of exhausted CD8^+^ T-cell function and increasing T-cell numbers are primary goals of ICB. Given the role that CD4^+^ T cells play in orchestrating immune responses, if feasible, restoration of exhausted CD4^+^ T-cell function by ICB may contribute significant clinical benefit in patients with tumors, either by improving direct CD4^+^ T-cell anti-tumor activity or increasing the helper functions of these cells. Exhaustion-associated markers on activated CD4^+^ T cells were upregulated during tumor progression, suggesting that these CD4^+^ T cells may also be responsive to PD-L1 blockade therapies. Our data demonstrate that co-treatment with ICB (αPD-L1) and tumor-specific CD4^+^ T-cell adoptive transfer can induce a tumor-specific CD4^+^ T lymphocyte proliferation response, which contributes to better tumor control. However, CD4^+^ T cells progress through multiple Th differentiation states in the context of tumors, leading to a unique requirement to consider functional changes in molecular programs within the context of specific Th differentiation states. Our data demonstrate that the majority of tumor-specific CD4^+^ T cells differentiated into Th1 cells, consistent with a previous report that the inhibiting of PD-L1 restored CD4^+^ Th1 cell amplification rapidly, enhancing CD4^+^ Th1 cytokine production and cytotoxic killing capacity during chronic infection in mice (48). Further, our finding that SMARTA cell efficacy was not reduced following FYT720 administration is of great importance. Given the role of lymph nodes in metastasis, they often need to be removed, which may compromise the efficacy of ICB treatment (49, 50). Our findings could provide an alternative approach for treating these patients.

Neoantigens have long been envisioned as optimal targets to induce anti-tumor immune responses. Recently, with the development of massively parallel sequencing for detecting all coding mutations within tumors, and machine learning approaches to predict which mutated peptides can bind autologous HLA molecules with high affinity, it has become more feasible to identify neoantigens. Studies of neoantigens have identified a peculiar phenomenon whereby peptides selected in silico for their ability to bind MHC class I largely yield CD4^+^ T-cell responses *in vivo* (51), potentially supporting the practical feasibility of CD4^+^ T-cell adoptive transfer therapy. Furthermore, human HLA transgenic mice can be immunized with human cancer antigens to generate highly tumor-reactive T-cells, which can then be used in cancer immunotherapy for humans. Infection-induced tumor-specific memory CD4^+^ T cells can prevent lung metastasis, inferring that the vaccine to prevent virus infection can also protect the host from tumorigenesis. In addition, the polyclonal tumor-reactive CD4^+^T cells also showed certain effect on the metastasis control, which give further evidence for the use of CD4^+^T cell ACT for tumor immunotherapy. These CD4^+^ T cells may act as helpers for cytotoxic CD8^+^ T cells through the licensing of dendritic cells *via* CD40/CD40L interactions (52). What’s more, CD4^+^ T cells have been proposed to have effector roles in the tumor microenvironment, including activation of local NK cells *via* secretion of effector cytokines, recruitment of CD8^+^ T cells by CXCL-10 release, and even HLA class II-dependent tumor cell killing (21); however, the precise mechanisms involved require further investigation. In contrast, the activated SMARTA cells couldn’t suppress solid tumor progression, which might be caused by their less infiltration into the tumor tissue and more severe exhaustion state within the solid tumor microenvironments, and a better understanding of the regulatory networks of antigen-specific CD4^+^ T cells between metastatic and solid tumor will be of great value in providing new insight for CD4^+^ T cell-based immunotherapies, which still need further investigation.

In conclusion, these data enhanced our fundamental understanding of the importance of tumor-reactive CD4^+^ T cells in the context of ACT and expanded the spectrum of CD4^+^ T cell-mediated anti-tumor immunity. A deeper exploration of the specific mechanisms and functional regulation of the CD4^+^ T cells may lead to more innovative and effective immunotherapies, which may provide an alternative way for the patients who are not respond to chemotherapy, radiotherapy or current immunotherapy approaches.

## Data Availability Statement

The raw data supporting the conclusions of this article will be made available by the authors, without undue reservation.

## Ethics Statement

The animal study was reviewed and approved by Animal Care and Use Committees of the Army Medical University.

## Author Contributions

LY, QH designed and oversaw experiments. QL, LW, HL, JW, ZW, JG, SW, LR, XS, QW, ZY, JT, ZL, LH performed experiments QL, LW, QH wrote the paper. LY supervised the study. All authors contributed to the article and approved the submitted version.

## Funding

This study was supported by grants from National Science and Technology Major Project (2021YFC2300502 to LY), National Science Foundation for Outstanding Young Scholars of China [NO. 82122028 to LX], the Chongqing Postdoctoral Science Foundation Project (NO.cstc2021jcyj-bshX0232 to QL), the National Natural Science Foundation of China(NO.319006 56 to ZW), and National Natural Science Foundation of China (No. 31900643 to QH)

## Conflict of Interest

The authors declare that the research was conducted in the absence of any commercial or financial relationships that could be construed as a potential conflict of interest.

## Publisher’s Note

All claims expressed in this article are solely those of the authors and do not necessarily represent those of their affiliated organizations, or those of the publisher, the editors and the reviewers. Any product that may be evaluated in this article, or claim that may be made by its manufacturer, is not guaranteed or endorsed by the publisher.
