# Supplementary material for: Tumor-Specific CD4+ T Cells Restrain Established Metastatic Melanoma by Developing Into Cytotoxic CD4– T Cells
Source: Front Immunol. 2022 Jun 16;13:875718. doi: 10.3389/fimmu.2022.875718 (PMC9243303; doi:10.3389/fimmu.2022.875718)
Supplement: Supplementary file 1 [file DataSheet_1.docx]

Supplementary Material

# Supplementary Figures and Tables

## Supplementary Figures

**
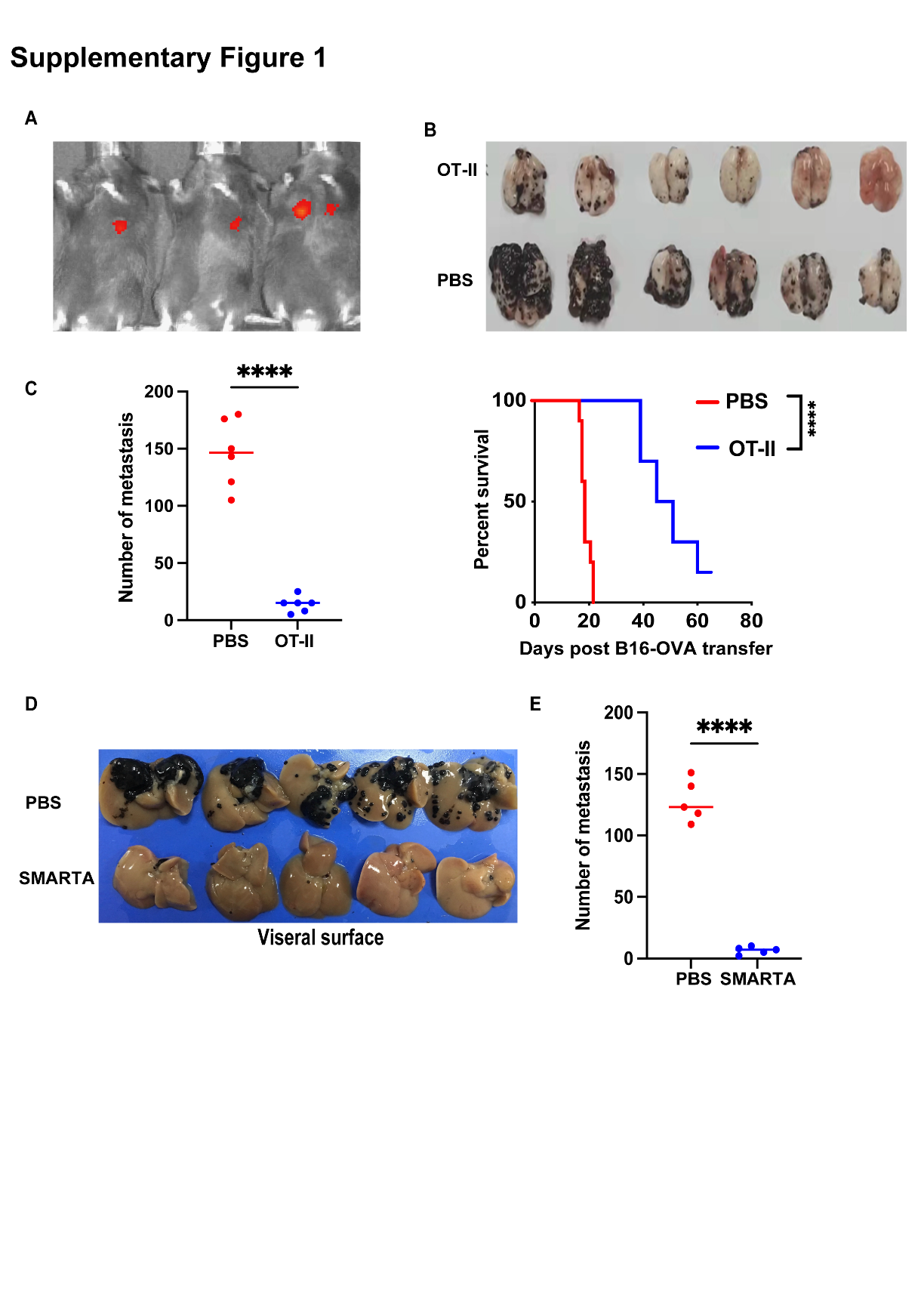
**

**Supplementary Figure 1.** Activated tumor-specific CD4^+^ T cells prevent lung and liver metastasis. **(A)** Bioluminescent imaging screening of tumor progression eight days after mice were inoculated i.v. with B16-GP cells. **(B, C)** C57BL/6J mice were injected with 5×10^5^ B16-OVA cells intravenously, followed by transferred with 2 × 10^6^ activated OT-II cells or PBS eight days later, and mice were sacrificed on Day 7 post-transfer. Representative image of lung samples harvested from tumor-bearing mice of PBS- or OT-II-treated group (B), the statistical analysis of metastatic foci numbers (n=6/group) and the survival cure (n=10/group) of the mice (C) are shown. **(D, E)** C57BL/6J mice (n=5/group) were intrasplenic injected with 5×10^5^ of B16-GP cells followed by splenectomy to develop the hepatic metastasis model. Tumor-bearing mice were treated i.v. with PBS or 2 × 10^6^ activated SMARTA cells on Day 4 post tumor cell inoculation and sacrificed on Day 17. Representative image of liver samples (visceral surface) harvested from tumor-bearing mice of PBS- or SMARTA-treated group (D) and the statistical analysis of metastatic foci numbers (E) are shown. Statistical differences are calculated by unpaired *t* test (C left and E, number of metastasis), Log-rank test (C right, survival curve). ****p* < 0.001, *****p* < 0.0001.

**
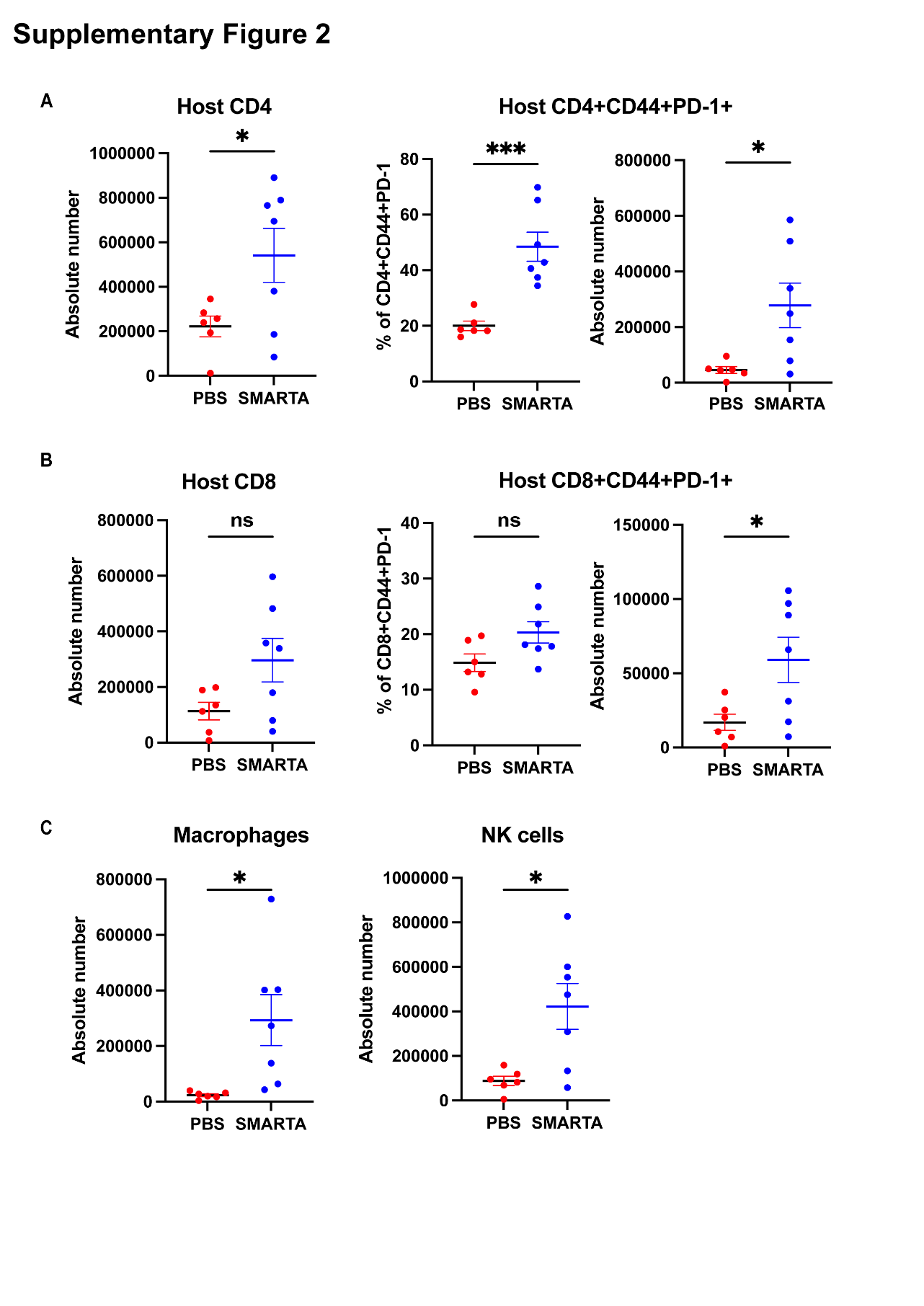
**

**Supplementary Figure 2.** The host immune responses against tumor metastases are boosted by activated tumor-specific CD4^+^ T cell transfer. The C57BL/6J mice were challenged i.v. by 5 × 10^5^ B16-GP cells and transferred with 2 × 10^6^ SMARTA cells (n=7) or PBS (n=6) eight days post B16-GP inoculation. Mice were sacrificed on Day 15. **(A)** The statistical analysis of the absolute number of the host CD4^+^ T cells, the frequency and absolute number of the host PD-1^+^ CD44^+^ CD4^+^ T cells in tumor-bearing mice. Cells analyzed are gated on live CD45.1^-^ cells. **(B)** The statistical analysis of the absolute number of the host CD8^+^ T cells as well as the frequency and absolute number of the host PD-1^+^ CD44^+^ CD8^+^ T cells in tumor-bearing mice. Cells analyzed are gated on live CD45.1^-^ cells. **(C)** The statistical analysis of the absolute number of the host macrophages and NK cells. Statistical differences are calculated by unpaired *t*-test. ns, not significant, **p* <0.05, *** *p* < 0.001. Data are presented as mean ± SEM.

**
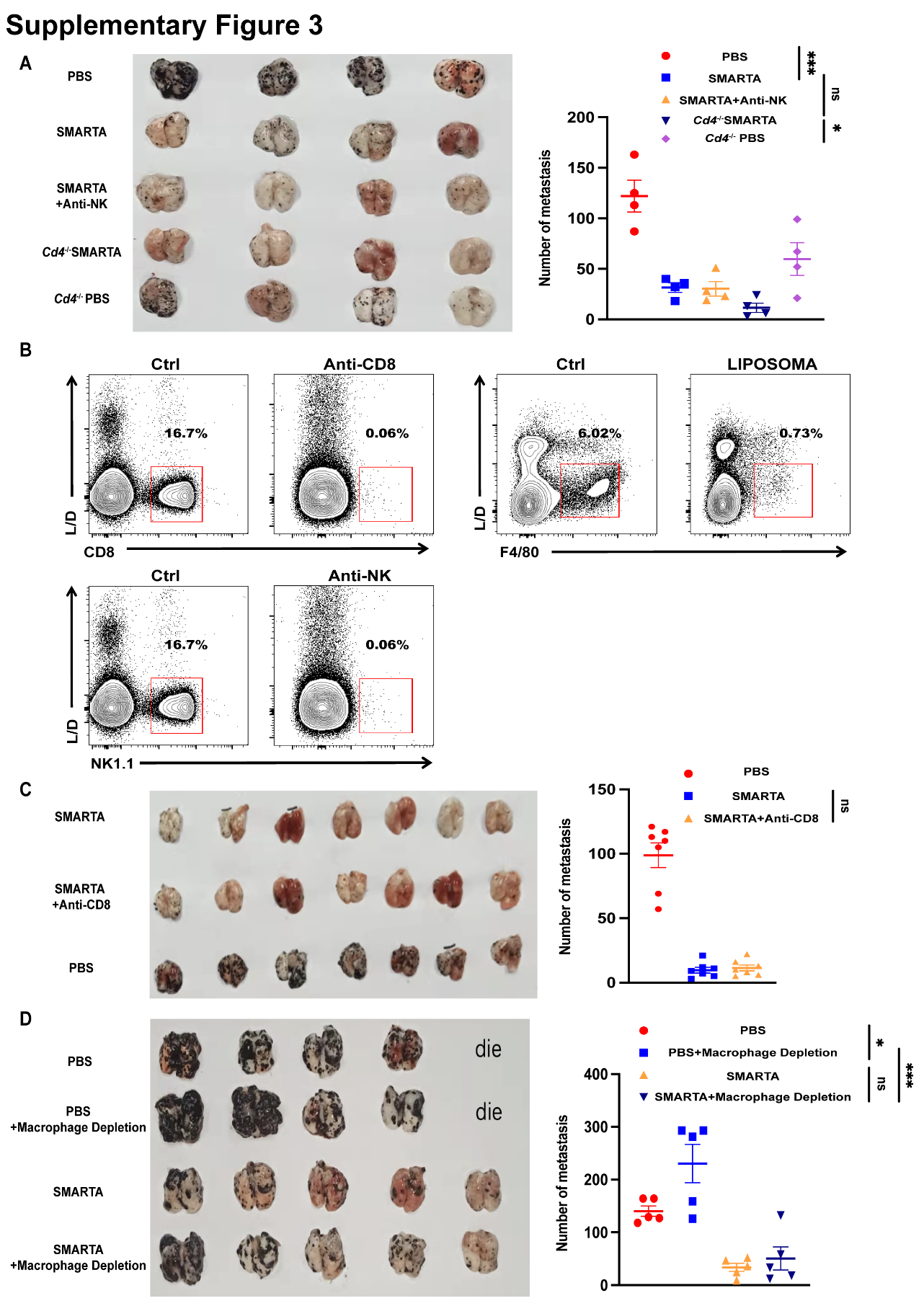
**

**Supplementary Figure 3.** Tumor-specific CD4^+^ T cell-mediated tumor rejection is independent of endogenous T, NK, and macrophage cells. **(A)** Image of lung samples harvested from the metastatic model in which tumor-bearing C57BL/6J mice received PBS or activated SMARTA cells only or combined with NK cell neutralizing antibody (anti-NK) treatment and the tumor-bearing *Cd4*^-/-^ mice were treated with SMARTA cells or PBS as mentioned above (n=4/group). Mice were sacrificed on Day 15 post tumor inoculation. The statistical analysis of the metastatic foci is shown beside. **(B)** Representative flow cytometry plots of CD8^+^ T cells, F4/80^+^ macrophages, and NK1.1^+^ NK cells in recipient tumor-bearing mice after the treatment of CD8, NK cell neutralizing antibodies or Clodronate Liposomes. Cells are gated on single cells. **(C)** Image of lung samples harvested from the metastatic model in which tumor-bearing C57BL/6J mice were transferred with activated SMARTA cells only or combined with CD8 neutralizing antibody (anti-CD8) treatment (n=7/group) and mice were sacrificed on Day 15. The statistical analysis of the metastatic foci is shown beside. **(D)** Image of lung samples harvested from tumor-bearing C57BL/6J mice which received PBS or activated SMARTA cells only or macrophage depleting treatment only, or in combination and were sacrificed on Day 15 post tumor cell inoculation (n=5/group). In the control group (PBS-treated), a mouse died on Day 14 and in the anti-macrophage group, a mouse died on Day 13. The foci of the dead mice were calculated as the same value as the most severe one. The statistical analysis of the metastatic foci is shown beside. Statistical differences are calculated by one-way ANOVA (A, C and D, number of metastasis). ns, not significant, **p* <0.05, *** *p* < 0.001. Data are presented as mean ± SEM.

**
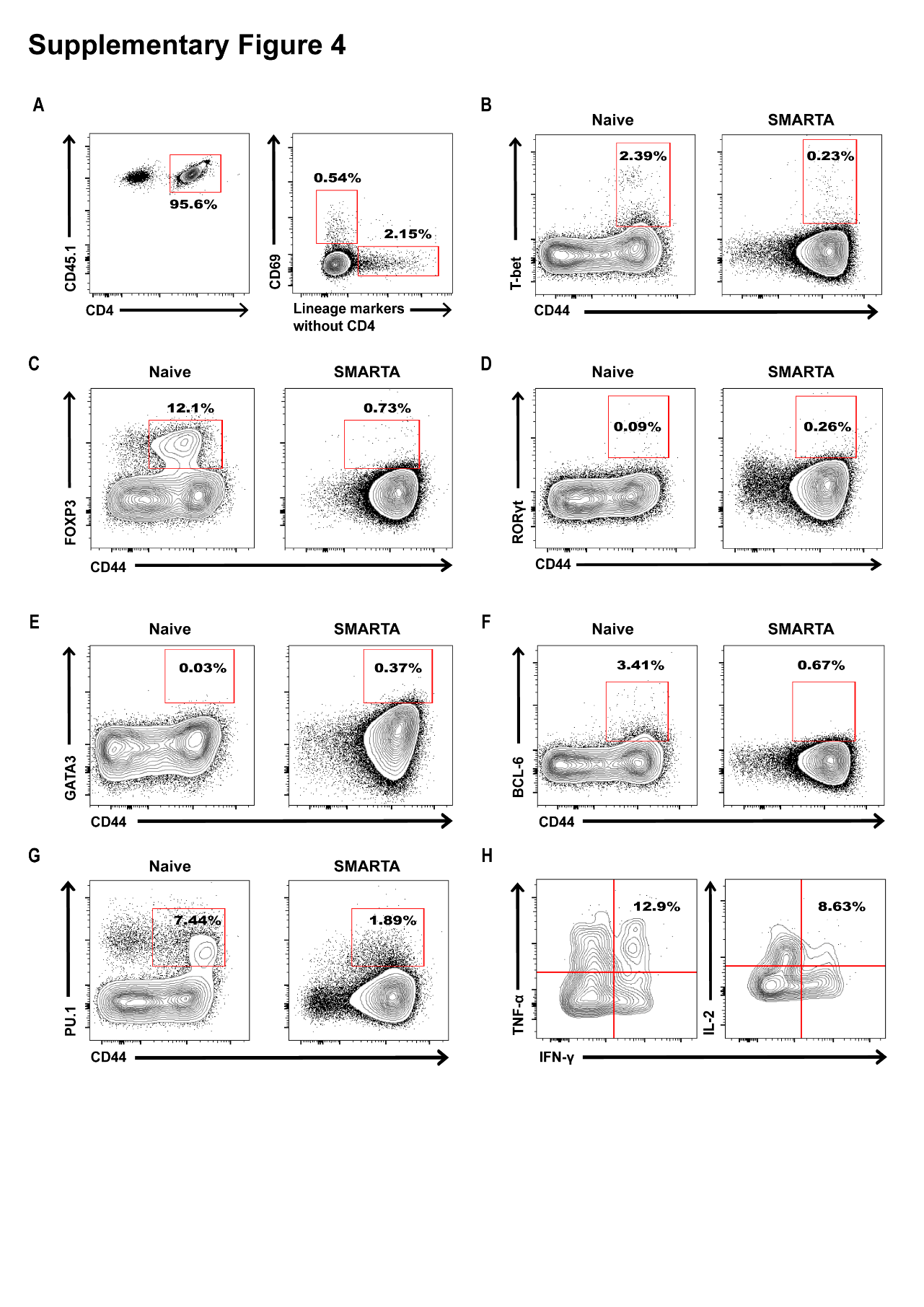
**

**Supplementary Figure 4.** The ex vivo activated SMARTA cells exhibited an unskewed phenotype. CD4^+^CD25^-^ cells were isolated from the splenocytes of CD45.1^+^ SMARTA mice and stimulated in vitro with anti-CD3 (1μg/mL)/anti-CD28 (1μg/mL) and 20 ng/mL IL-2 for 48h, followed by cultured with IL-2 for another 7 days. The activated and expended cells after culture were analyzed prior to transfer. **(A)** left panel: the flow cytometry plots of co-expression of CD4 and CD45.1 in the activated cells. Right panel: other lineage markers (CD8, B220, CD19, NK1.1, F4/80, CD11b, CD11c, TER-119) stained by biotin-conjugated antibodies with streptavidin and the expression of CD69 in the activated cells. Cells are gated on live cells. **(B-G)** The flow cytometry plots showing the expression of CD4^+^ T cell lineage-related transcription factors, including T-bet (B), FOXP3 (C), RORγt (D), GATA3 (E), BCL-6 (F), and PU.1 (G) in SMARTA cells as indicated in A. Cells are gated on live CD45.1^+^CD4^+^T cells. Naïve CD4^+^ T cells from C57BL/6J mice splenocytes were analyzed as the control. The numbers in the plots represent the percentage of gated positive populations. **(H)** The flow cytometry plots showing the expression of TNF-α, IFN-γ and IL-2 in activated SMARTA cells after PMA/ionomycin re-stimulation.

**
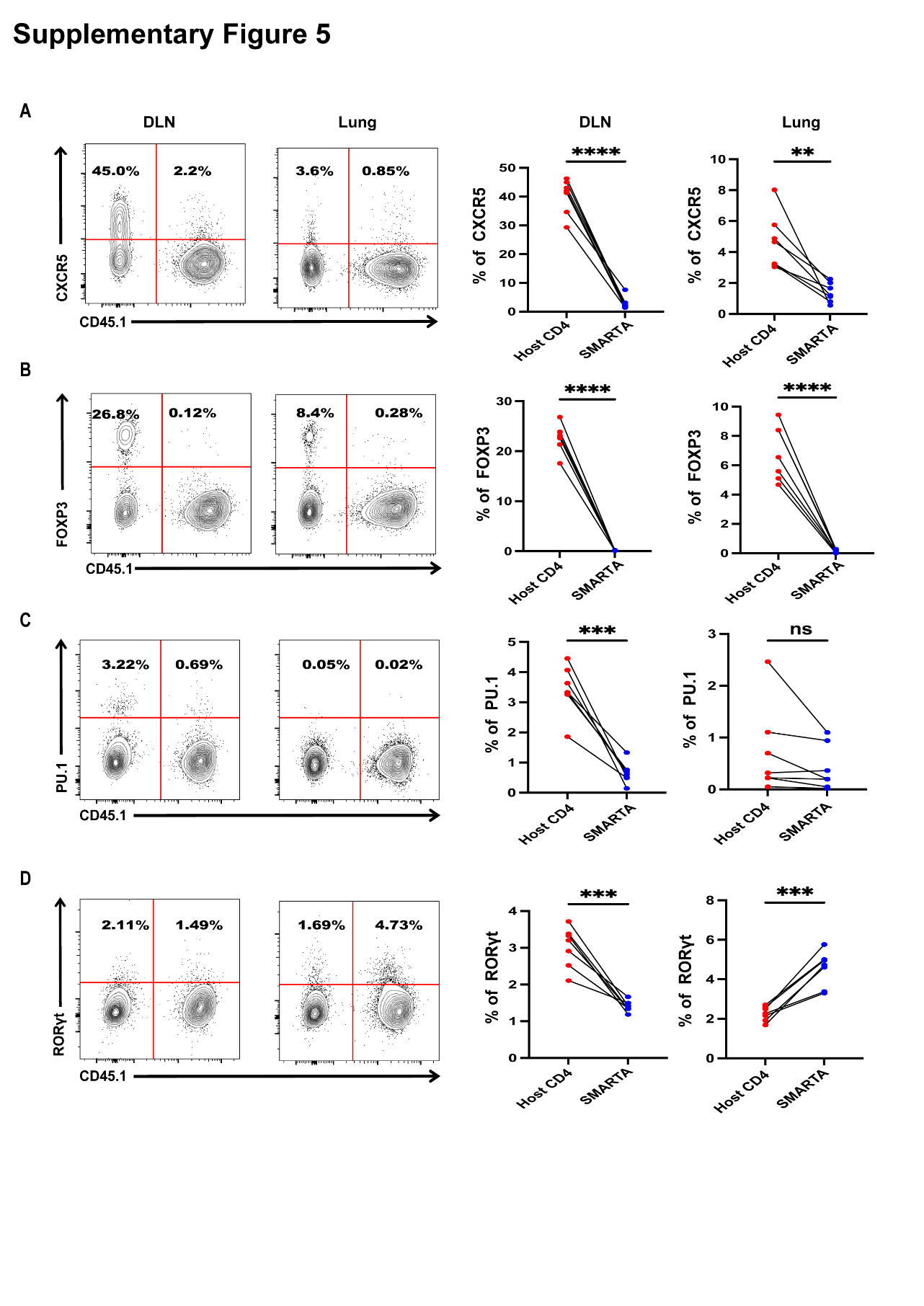
**

**Supplementary Figure 5.** Transferred SMARTA cells didn’t differentiate into other lineage subtypes of CD4^+^ T cells. B16-GP tumor-bearing C57BL/6J mice (n=7) were treated with 2 × 10^6^ activated SMARTA cells on Day 8 and sacrificed seven days later. Cells are gated on live CD4^+^CD44^+^cells, and numbers in the figures of flow cytometry plots indicate the percentages of gated populations accounting for CD45.1^-^(left quadrant, host CD4^+^ T cells) or CD45.1^+^ cells (right quadrant, SMARTA). **(A)** FACS data of CXCR5 expression in lymphocytes from DLN and lung. The frequencies of CXCR5^+^ cells in DLN and lung are summarized beside. **(B)** FACS data of FOXP3 expression in lymphocytes from DLN and lung. The frequencies of FOXP3^+^ cells in DLN and lung are summarized beside. **(C)** FACS data of PU.1 expression in lymphocytes from DLN and lung. The frequencies of PU.1^+^ cells in DLN and lung are summarized beside. **(D)** FACS data of RORγt expression in lymphocytes from DLN and lung. The frequencies of RORγt ^+^ cells in DLN and lung are summarized beside. Statistical differences are calculated by paired *t* test. ns, not significant, ** *p* <0.01, ****p* < 0.001, *****p* < 0.0001.

**
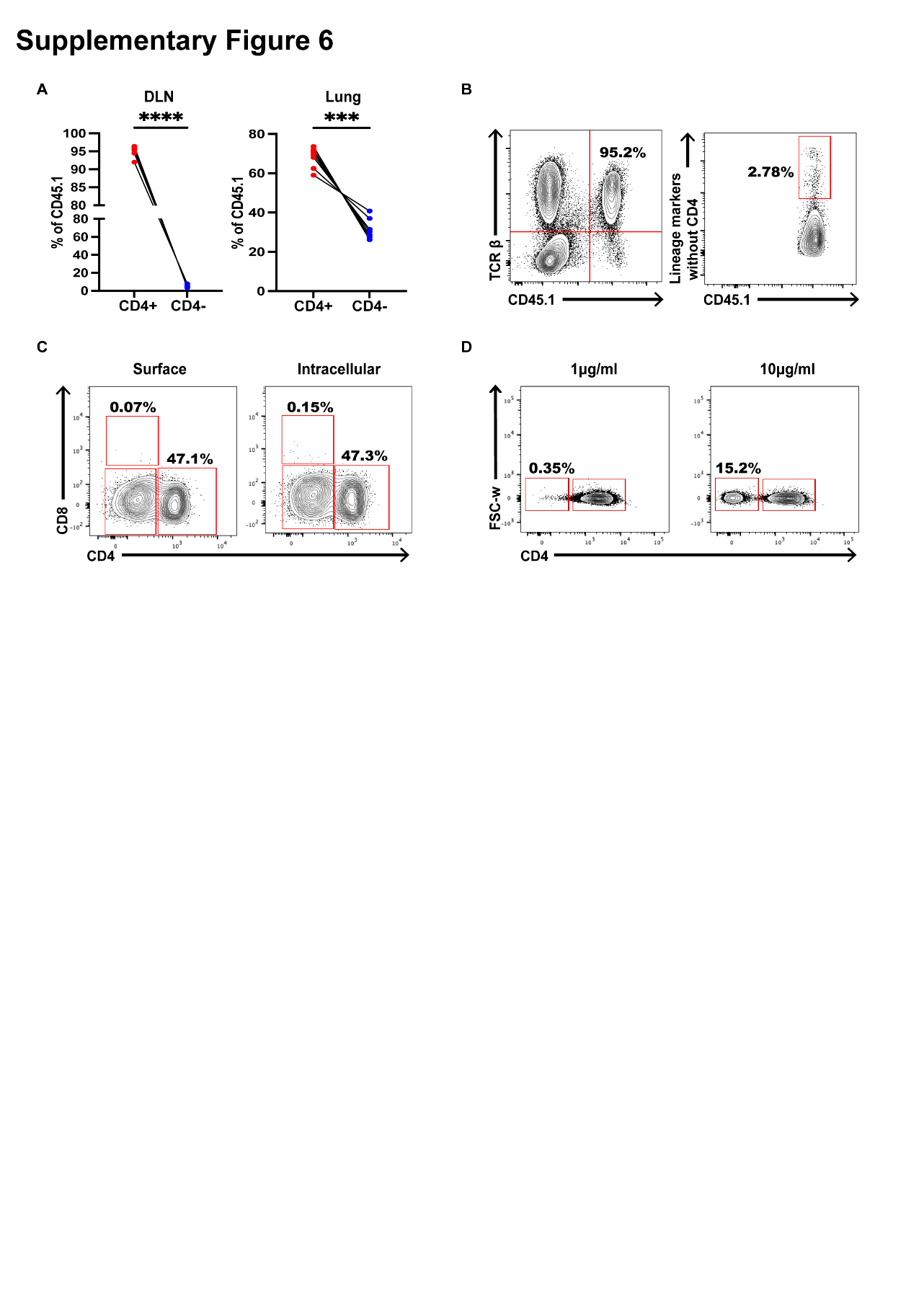
**

**Supplementary Figure 6.** CD4^+^ T cells differentiate into CD4^-^ T cells in vivo. Naïve CD4^+^SMARTA cells were isolated and purified from spleens of CD45.1^+^SMARTA mice and activated and cultured in vitro. The C57BL/6J mice were challenged i.v. by 5 × 10^5^ B16-GP cells and transferred with 2 × 10^6^ activated SMARTA cells eight days post B16-GP inoculation. Mice were sacrificed on Day 15. **(A)** Statistical analysis of the percentages of CD4^+^ and CD4^-^ SMARTA cells in DLN and lung tissue as shown in Figure 2C (n=7/group). **(B)** Left panel: representative flow cytometry plots of co-expression of CD45.1 and TCRβ in lymphocytes harvested from lung tissue of tumor-bearing mice. Cells are gated on live cells. The number in the figures indicates the percentage of TCRβ^+^CD45.1^+^ population accounting for CD45.1^+^ SMARTA cells. Right panel: representative flow cytometry plots showing CD45.1^+^ SMARTA cells’ expression of other lineage markers (CD8, B220, CD19, NK1.1, F4/80, CD11b, CD11c, TER-119) stained by biotin-conjugated antibodies with streptavidin. Cells are gated on live CD45.1^+^ cells. **(C)** The representative flow cytometry plots of CD4 and CD8 expression in lymphocytes of the lung from tumor-bearing mice on Day 15 after SMARTA cell transfer, examined through surface staining alone or in combination with intracellular staining using Foxp3/Transcription Factor Staining Kit. Cells are gated on Live CD45.1^+^ cells. **(D)** FACS data analyzing the downregulation of CD4 by SMARTA cells after stimulation in vitro with anti-CD3 of different concentrations (1 μg/mL or 10 μg/mL) and 1 μg/mL anti-CD28 for 48 hours coupled with 20 ng/mL IL-2 and cultured for another six days only with 20 ng/mL IL-2. Cells are gated on live CD45.1^+^cells. Statistical differences are calculated by paired *t* test. ****p* < 0.001, *****p* < 0.0001.

**
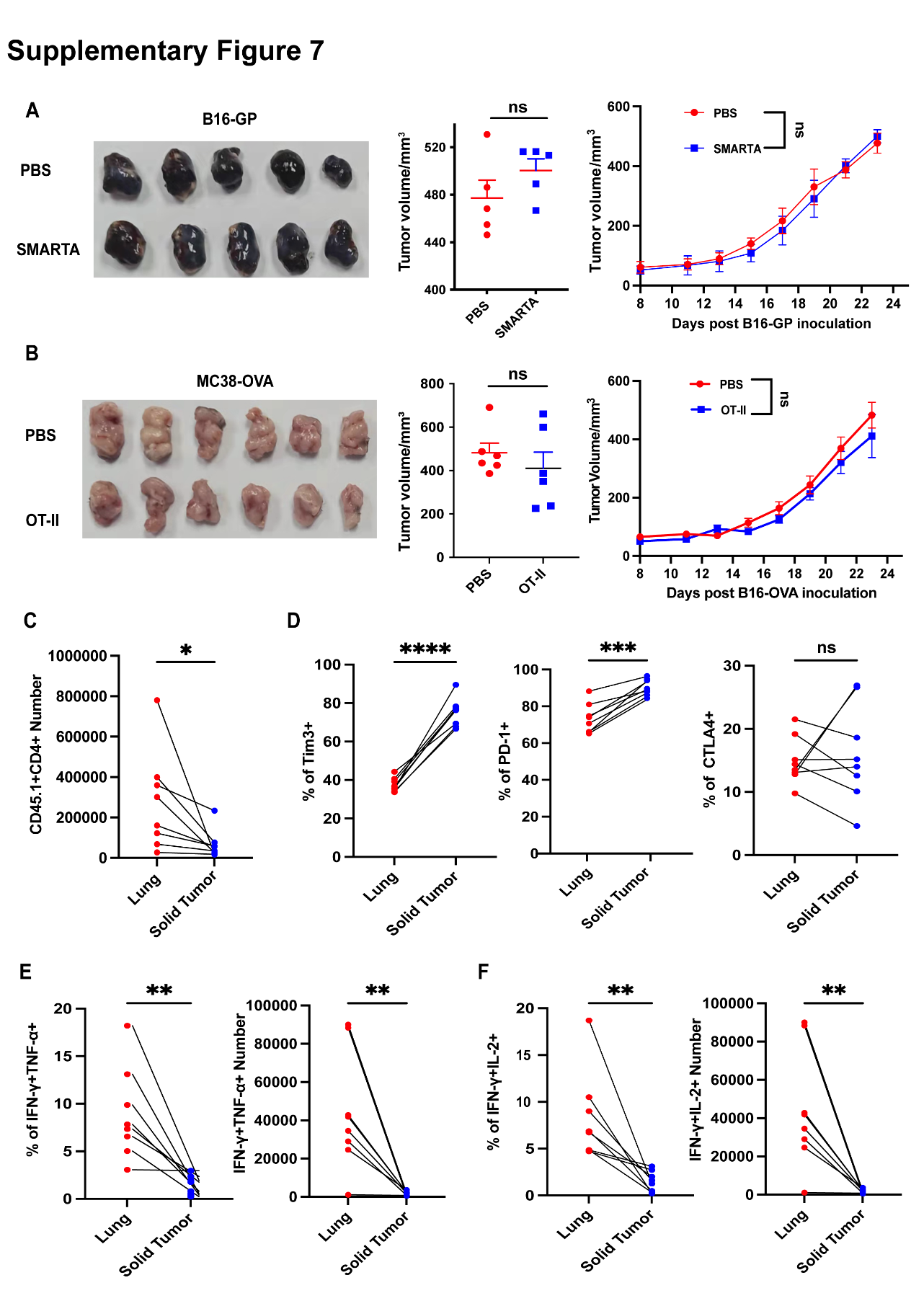
**

**Supplementary Figure 7.** Activated tumor-specific CD4^+^ T cells could not control solid tumors. **(A)** Picture of B16-GP tumor samples harvested from in situ B16-GP tumor model in which C57BL/6J mice were implanted with B16-GP tumor cells subcutaneously and treated with PBS or 2 × 10^6^ activated SMARTA cells on Day 8 post tumor cell inoculation and sacrificed on Day 25 (n=5/group). The statistical analysis of B16-GP tumor volume on Day 25 and the tumor growth curve of the PBS- and SMARTA-treated group are shown beside. **(B)** Picture of MC38-OVA tumor samples harvested from in situ MC38-OVA tumor model in which C57BL/6J mice were implanted with MC38-OVA tumor cells subcutaneously and treated with PBS or 2 × 10^6^ activated OT-II cells on Day 8 post tumor inoculation and were sacrificed on Day 25 (n=6/group). The statistical analysis of tumor volume of MC38-OVA on Day 25 post tumor implantation and the tumor growth curve of the PBS- and OT-II-treated group are shown beside. **(C-F)** B16-GP cells were inoculated into C57BL/6J mice via tail vein and subcutaneous injection simultaneously to build a model with both in situ and metastatic tumors (n=8). Tumor-bearing mice were transferred with 2 × 10^6^ activated CD45.1^+^SMARTA cells on Day 8 post tumor cell challenge and sacrificed on Day 15. Transferred SMARTA cells were recovered from mice lung and solid tumor tissue and analyzed by flow cytometry. The absolute number of CD45.1^+^CD4^+^ T cells (C), the frequencies of Tim3^+^, PD-1^+^ and CTLA4^+^ SMARTA cells (D), the frequencies and absolute numbers of IFNγ^+^TNF-α^+^ (E) and IFNγ^+^IL-2^+^ (F) SMARTA cells in the lung tissue and solid tumor mass were summarized. Statistical differences are calculated by unpaired *t* test (A, B tumor volume), two-way ANOVA with a *post hoc* Turkey test (A, B, tumor growth curve), or paired *t* test (C-F). ns, not significant, **p* <0.05, ** *p* <0.01, ****p* < 0.001, *****p* < 0.0001.

## Supplementary Tables

**Supplementary table 1. Antibodies and reagents used in flow cytometry**

| **Antibody** | **Clone** | **Dilution** | **Provider** |
| --- | --- | --- | --- |
| CD4 Brilliant Violet 510 | RM4-5 | 1:200 | Biolegend |
| CD4 PerCP | RM4-5 | 1:200 | Biolegend |
| CD8 PerCP | 53-6.7 | 1:200 | Biolegend |
| CD8 Brilliant Violet 510 | 53-6.7 | 1:200 | Biolegend |
| CD3e FITC | 145-2C11 | 1:200 | eBioscience |
| CD44 PE/Cyanine7 | IM7 | 1:200 | eBioscience |
| CD44 PerCP | IM7 | 1:200 | eBioscience |
| CD45.1 APC | A20 | 1:200 | Biolegend |
| CD45.1 PerCP | A20 | 1:200 | Biolegend |
| CD45.1 PE/Cyanine7 | A20 | 1:200 | Biolegend |
| CD45.1 FITC | A20 | 1:200 | Biolegend |
| CD45.2 FITC | 104 | 1:200 | Biolegend |
| CD45.2 APC | 104 | 1:200 | Biolegend |
| TCR Va2 PE | B20.1 | 1:100 | Biolegend |
| LIVE/DEAD Fixable Near-IR Dead Cell Stain Kit | / | 1:200 | Life Technologies |
| F4/80 biotin | BM8 | 1:200 | Biolegend |
| NK1.1 biotin | PK136 | 1:200 | Biolegend |
| CD45R/B220 biotin | RA3-6B2 | 1:200 | Biolegend |
| CD11b biotin | M1/70 | 1:200 | Biolegend |
| CD11c biotin | N418 | 1:200 | Biolegend |
| Gr-1 biotin | RB6-8C5 | 1:200 | Biolegend |
| TER-119 biotin | TER-119 | 1:200 | Biolegend |
| CD25 biotin | PC61 | 1:200 | Biolegend |
| GITR biotin | DTA-1 | 1:100 | Biolegend |
| T-bet PE | 4B10 | 1:100 | Biolegend |
| T-bet APC | 4B10 | 1:100 | Biolegend |
| PU.1 PE | 7C2C34 | 1:100 | Biolegend |
| RORγt APC | B2D | 1:100 | eBioscience |
| Foxp3 PE | FJK-16s | 1:100 | eBioscience |
| Foxp3 APC | FJK-16s | 1:100 | eBioscience |
| CXCR5 | 2G8 | 1:100 | BD Biosciences |
| Biotin Goat Anti-Rat IgG | 112-065-143 | 1:300 | Jackson Immunoresearch |
| Streptavidin PB | 25-4317-82 | 1:100 | eBioscience |
| PD-1 PE | RMP1-30 | 1:100 | eBioscience |
| PD-1 FITC | RMP1-30 | 1:100 | eBioscience |
| CTLA4 PE | UC10-4B9 | 1:100 | Biolegend |
| Tim3 PE | RMT3-23 | 1:100 | BD Biosciences |
| Tim3 BV421 | RMT3-23 | 1:100 | BD Biosciences |
| Ki-67 FITC | B56 | 1:100 | BD Biosciences |
| Ki-67 PE | B56 | 1:100 | BD Biosciences |
| Bcl2 PE | / | 1:100 | BD Biosciences |
| CD69 PE/Cyanine7 | H1.2F3 | 1:100 | BD Biosciences |
| CD69 PE | H1.2F3 | 1:100 | BD Biosciences |
| CD103 PE | 2E7 | 1:100 | Biolegend |
| CD103 Brilliant Violet 421 | 2E7 | 1:100 | Biolegend |
| IFN-γ Brilliant Violet 421 | XMG1.2 | 1:100 | BD Biosciences |
| TNF-α PE/Cyanine7 | MP6-XT22 | 1:100 | BD Biosciences |
| IL-2 PE | JES6-5H4 | 1:100 | Biolegend |
| Granzyme A PE | 3G8.5 | 1:100 | Biolegend |
| Granzyme B APC | QA16A02 | 1:100 | Biolegend |
| MHC-Ⅱ APC | AF6-120.1 | 1:100 | Biolegend |
| MHC-Ⅰ PE | AF6-88.5.5.3 | 1:100 | eBioscience |
| TCR β chain PE/Cyanine7 | H57-597 | 1:100 | Biolegend |

**Supplementary table 2. Blockade antibodies**

| **Antibody target/Reagent** | **Clone No.** | **Dosage/Dilution** | **Provider** |
| --- | --- | --- | --- |
| Anti-PD-L1 | 10F.9G2 | 150μg/mouse (In vivo) | BioXCell |
| Anti-CD8 | YTS-169.4 | 50μg/mouse (In vivo) | BioXCell |
| Anti-NK | PK136 | 50μg/mouse (In vivo) | Biolegend |
| Anti-CD3ε | 145-2C11 | 1μg/ml (In vitro) | Biolegend |
| Anti-CD28 | 37.51 | 1μg/ml (In vitro) | Biolegend |
